# Association of Periodontal Condition With Impaired Glucose Tolerance: Results of a 15‐Year Follow‐Up Study

**DOI:** 10.1002/cre2.70023

**Published:** 2024-10-13

**Authors:** Ville Myllymäki, Pekka Ylöstalo, Anna Liisa Suominen, Matti Knuuttila, Ulla Rajala, Sirkka Keinänen‐Kiukaanniemi, Sirpa Anttila, Tuomas Saxlin

**Affiliations:** ^1^ Institute of Dentistry University of Eastern Finland Kuopio Finland; ^2^ Department of Medicine University of Helsinki Helsinki Finland; ^3^ Medical Research Center Oulu Oulu University Hospital and University of Oulu Oulu Finland; ^4^ Department of Oral and Maxillofacial Surgery Oulu University Hospital Oulu Finland; ^5^ Research Unit of Population Health, Faculty of Medicine University of Oulu Oulu Finland; ^6^ Odontology Education Unit Kuopio University Hospital Kuopio Finland; ^7^ Department of Public Health and Welfare Finnish Institute for Health and Welfare Helsinki Finland; ^8^ Unit of Primary Health Care Oulu University Hospital Oulu Finland; ^9^ Wellbeing Services County of North Ostrobothnia Pyhäjärvi Finland; ^10^ Dental Training Clinic, Oral Health Care, Wellbeing Services County of North Ostrobothnia Finland

**Keywords:** cohort study, impaired glucose tolerance, oral health, periodontal condition, periodontitis

## Abstract

**Objectives:**

The aim of this study was to investigate whether periodontal condition is associated with the development of impaired glucose tolerance (IGT).

**Material and Methods:**

This study was based on a subpopulation of a cohort of persons born in 1935 and living in Oulu, Finland, on October 1, 1990. The participants were normoglycemic (no previously diagnosed diabetes mellitus and a 2‐h oral glucose tolerance test [OGTT] blood glucose < 7.8 mmol/L) in the baseline examinations (1990–1992) and had fasting blood glucose < 7.0 mmol/L in the follow‐up examinations (2007–2008) (*n* = 225). The outcome was IGT on follow‐up, measured by a blood glucose level of ≥ 7.8 mmol/L after OGTT. The exposure was the periodontal condition at baseline categorized into four groups: 0, 1–6, ≥ 7 sites with deepened (≥ 4 mm) periodontal pockets, and edentulousness.

**Results:**

A total of 23% of the participants developed IGT. The adjusted incidence rate ratios with 95% confidence intervals (CI) for dentate participants with 1–6 sites and ≥ 7 sites with deepened periodontal pockets, and edentate participants (reference category dentate participants without deepened periodontal pockets) were 1.5 (95% CI, 0.6–4.0), 1.8 (95% CI, 0.7–4.4), and 1.6 (95% CI, 0.6–4.0), respectively.

**Conclusions:**

Poor periodontal condition may predispose individuals to IGT; however, further studies on this matter are warranted.

## 1 Introduction

Impaired glucose tolerance (IGT) is a component of the prediabetic condition along with impaired fasting glucose (IFG). IGT is defined as a fasting blood glucose level < 7.0 mmol/L and a blood glucose level of 7.8–11.1 mmol/L 2 h after ingestion of a 75‐g oral glucose load (WHO and IDF). Although reversible, IGT is a state with an increased risk of progressing to diabetes mellitus (DM). Several factors have been found that increase the risk of developing IGT, including obesity (Greiner et al. 2020; Mansourian et al. 2018; Saleem et al. 2019), physical inactivity (Saleem et al. 2019), high age (Greiner et al. 2020; Mansourian et al. 2018; Saleem et al. 2019), high‐fat intake (Mansourian et al. 2018), hypertension (Greiner et al. 2020; Mansourian et al. 2018; Saleem et al. 2019; Díaz‐Redondo et al. 2015), a low high‐density lipoprotein level (Díaz‐Redondo et al. 2015), and parental DM (Greiner et al. 2020).

Another factor suggested to be associated with IGT is periodontitis, an inflammatory disease of the tooth‐supporting apparatus (Song et al. 2021; Arora et al. 2014; Choi et al. 2011; Saito et al. 2004), and the low‐level systemic inflammation induced by periodontitis (Nesse et al. 2008) has been suggested to be the mechanistic link between these conditions (Nesse et al. 2009). Long‐standing inflammation in the tissues surrounding the teeth can produce an acute‐phase response by the liver, which includes the release of interleukin‐6 and the activation of peripheral neutrophils to release oxygen radicals, thus creating a peripheral oxidative stress response (Meyle and Chapple 2015; Allen et al. 2011; Bullon et al. 2009; Chapple and Matthews 2007). Although periodontitis has been found to be associated with IGT in several studies, conflicting findings also exist (Kowall et al. 2015; Marugame et al. 2003). Furthermore, although the role of IGT in the progression of periodontitis has been investigated (Andriankaja et al. 2022), longitudinal studies focusing on the association of poor periodontal condition with the development of IGT are practically non‐existent. One exception is a study by Song et al. (2021), which reported an association between a poor periodontal condition and a prediabetic condition measured by IFG, but not by IGT. In addition to inconsistency, methodological differences in studies regarding the association of poor periodontal condition with IGT warrant additional research on the topic.

The aim of this cohort study was to investigate whether periodontal condition, measured as the number of sites with deepened (≥ 4 mm) periodontal pockets and edentulousness, is associated with the development of IGT over a follow‐up period of approximately 15 years in a study population with normal glucose metabolism at baseline. The hypothesis was that poor periodontal condition is associated with disturbed glycemic control measured in terms of IGT.

## 2 Methods

**Figure 1:**
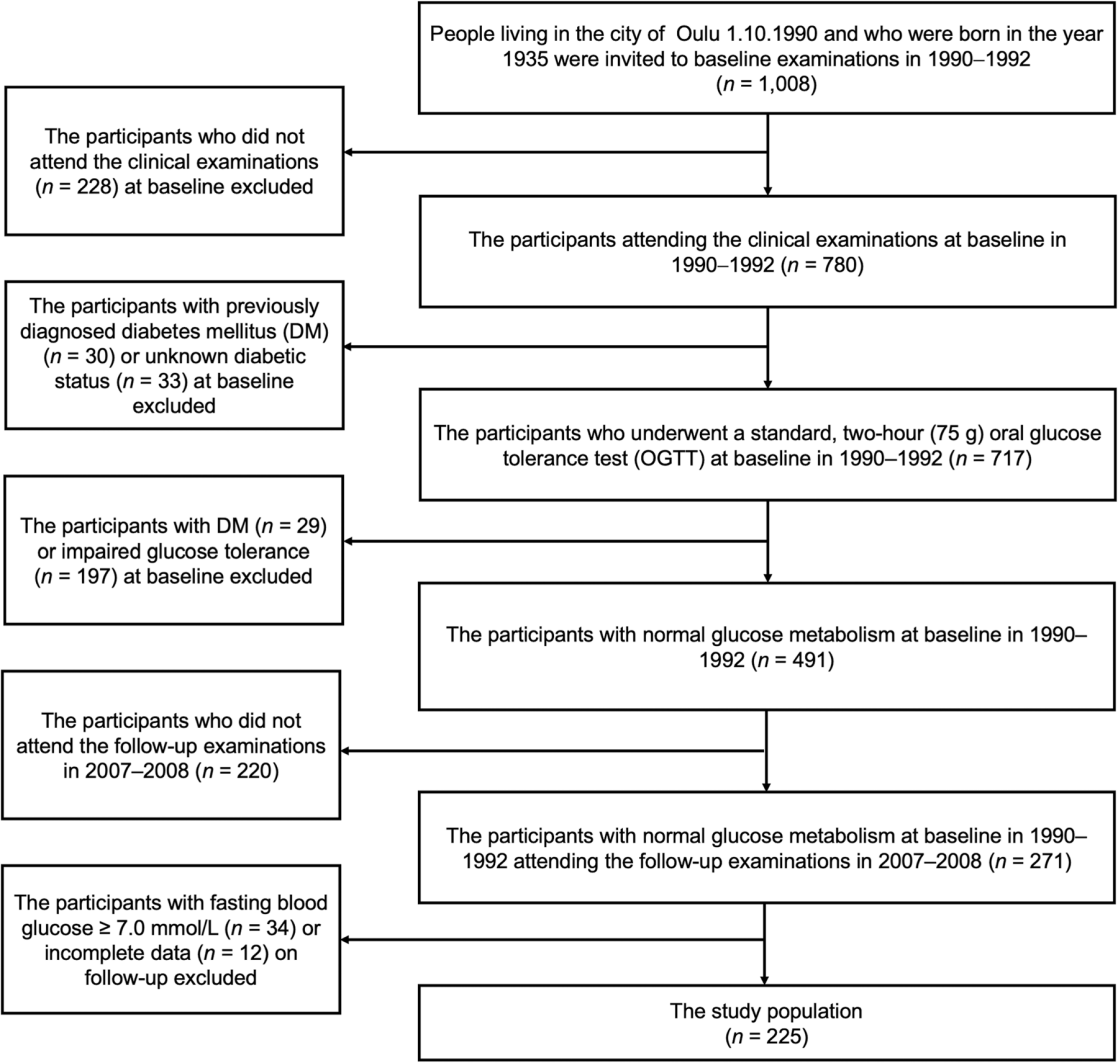
Flowchart of the formation of the study population.

This study was based on a cohort of persons born in 1935 who were inhabitants of Oulu, Finland, on October 1, 1990. The study population was examined in two phases: the baseline examinations between 1990 and 1992 and the follow‐up examinations between 2007 and 2008. The data were collected using postal questionnaires, interviews, clinical examinations, and laboratory tests. Altogether, 1,008 participants were invited at baseline, of whom 780 participated in the clinical examinations, including oral examinations. Of these 780 participants, 30 had previously diagnosed DM, and the diabetic status of 33 participants was unconfirmed (Rajala et al. 1995). Furthermore, 717 of the 780 participants of the baseline clinical examinations participated in a standard, 2‐h (75 g) OGTT. At baseline, venous whole blood samples were utilized for the fasting blood glucose and capillary samples for the 2‐h post‐load blood glucose determination using the hexokinase‐glucose‐6‐phosphate dehydrogenase method (Merck Diagnostica, Darmstadt, Germany) (Rajala et al. 1995). Based on the baseline OGTTs, 29 participants had previously undiagnosed DM according to the contemporary criteria set by the World Health Organization (WHO) (2‐h post‐load blood glucose value ≥ 11.1 mmol/L) (Rajala et al. 1995; World Health Organization 1985). Moreover, 197 participants were prediabetics (IGT) according to the same WHO criteria (fasting blood glucose value < 6.7 mmol/L and 2‐h post‐load blood glucose value 7.8–11.1 mmol/L) (Rajala et al. 1995; World Health Organization 1985). Of the 491 normoglycemic participants at baseline (confirmed by OGTT), 271 participated in the follow‐up examinations. Of these, the participants with a fasting blood glucose level ≥ 7.0 mmol/L on follow‐up (*n* = 34)—based on the criteria of IGT by WHO and International Diabetes Federation (IDF)—as well as those with a lack of data about their smoking status and history (*n* = 6) or the hereditary risk of DM (*n* = 4), were excluded. In addition, two participants were excluded for not participating in OGTT at follow‐up. Thus, the final study population consisted of 225 participants who had normal glucose metabolism at baseline, and who participated in the clinical oral examinations at baseline and OGTT on follow‐up. The formation of the study population is depicted in Figure 1. The Oulu 1935 cohort study was conducted in full accordance with the ethical principles of the Helsinki Declaration (as revised in 1975) (Declaration of Helsinki 1976). Informed consent was received from all participants, and the study was approved by the Ethics Committee of the Faculty of Medicine at the University of Oulu, Oulu, Finland.

The outcome variable of this study was IGT on follow‐up in 2007–2008. This was defined as a 2‐h post‐load blood glucose value > 7.8 mmol/L in OGTT, based on recommendations from the WHO & IDF (2006). Venous blood samples were drawn into containers with glycolytic inhibitors and were centrifuged to separate the plasma. The glucose concentration was determined using the hexokinaseglucose‐6‐phosphate dehydrogenase method (Cederberg et al. 2010). IGT was treated as a dichotomous variable (no/yes) in the statistical analyses.

The exposure variable of this study was periodontal condition, depicted as the number of sites with deepened (≥ 4 mm) periodontal pockets, measured as a part of the clinical oral examinations at baseline in 1990–1991. Probing pocket depths were measured, and depths of ≥ 4 mm were registered, from the mesial, distal, buccal, and lingual surfaces of the teeth. The periodontal examinations were performed by two identically instructed examiners whose probing techniques were calibrated. The intra‐examiner agreement in the diagnosis of deepened periodontal pockets was 96.7% with a kappa statistic of 0.80 for examiner 1, and 95.3% with a kappa statistic of 0.78 for examiner 2 (Sakki et al. 1995). In statistical analyses, periodontal condition at baseline was treated as a categorized variable. The categorization was carried out as follows: periodontally healthy participants (dentate participants without deepened periodontal pockets), participants with 1–6 sites with deepened periodontal pockets, participants with ≥ 7 sites with deepened periodontal pockets, and edentate participants. The cut‐off point was determined based on the median value of the number of sites with deepened periodontal pockets.

Other variables and potential confounders of this study included gender, the level of education (baseline data), yearly household income (baseline data), the hereditary risk of DM (follow‐up data), physical activity (baseline data), dietary habits (baseline data), smoking status (follow‐up data), body mass index (BMI) (follow‐up data), and the absolute change in BMI during the follow‐up period, the serum levels of triglycerides and high‐density lipoprotein cholesterol (HDL‐C) (follow‐up data), and arterial hypertension (follow‐up data).

Information on the level of education was obtained through a postal questionnaire before the baseline examinations and was categorized into three categories: basic (vocational school or lower), intermediate (graduated from a vocational college or university of applied sciences), and higher (graduated from a university) following the International Standard Classification of Education (UNESCO 1997). Information on the yearly household income was also obtained from the postal questionnaire and was categorized as follows: less than 20,400€, 20,400–30,599€, 30,600–40,800€, and more than 40,800€.

The hereditary risk of DM was based on the occurrence of DM in the immediate family. This information was obtained through an interview conducted during the follow‐up examinations using the following question: “Has anyone in your immediate family or any relatives been diagnosed with diabetes mellitus?” The answer options were the following: “No,” which was categorized as low hereditary risk; “Yes, diabetes mellitus was diagnosed among grandparents, siblings of parents or cousins (but not among own parents, siblings or children),” and “Yes, diabetes mellitus was diagnosed among parents, siblings or own children,” which were categorized as an increased hereditary risk.

Information on physical activity was obtained through an interview conducted during the baseline examinations. Physical activity was categorized as low if the participant walked or cycled less than 15 min on the way to work and exercised only once a week or less in their free time. In other cases, physical activity was categorized as high.

Information on dietary habits was obtained from responses to the following three questions of the interview during the baseline examinations: “How often do you eat vegetables, fruits, or root crops?” with the response options being “Daily” and “Less than once a week”; “What is the fat content of the butter or margarine you use on bread?” with the response options being “Nothing or light margarine” and “Butter”; and “What is the fat content of the drink you have with meals?” with response options being “Non‐fat (for instance water, fat‐free milk or sour milk),” “Low‐fat (light milk or sour milk),” and “High‐fat (fatty milk).” Dietary habits were categorized into three categories as follows: “Healthy diet” if the participant chose the healthiest response options for all three questions; “Moderately healthy diet” if the participant chose one unhealthy option; and “Unhealthy diet” if the participant chose more than one unhealthy option.

Information on the smoking status was obtained through an interview conducted during the follow‐up examinations using questions about whether the participant had ever smoked regularly (defined as smoking almost every day and for at least 1 year in their lifetime [“Yes”/“No”]), about the current smoking status (“Regularly”/“Occasionally”/“No smoking”), and, if the participant had smoked previously, at what age smoking had ceased. Based on these questions, the participants' smoking status was categorized into three categories: never smoker, former smoker, and current smoker. Participants were categorized as never smokers if the answer to the first question was “No” and as former smokers if the third question was answered. Participants were deemed as current smokers if the answer to the second question was “Regularly.” For the multivariate analyses, the categories never smoker and former smoker were combined as one category (non‐smoker).

Height and weight in light clothing were measured during the clinical examination both at baseline and follow‐up by a trained research nurse. BMI was calculated based on these measurements. BMI on follow‐up and the absolute change in BMI during the follow‐up period were used as continuous variables in the multivariate analyses.

The serum levels of triglycerides and HDL‐C were determined from venous blood after a 10‐ to 12‐h fast in the follow‐up examinations using a manual enzymatic CHOD‐PAP method (Boehringer Mannheim, Mannheim, Germany). HDL‐C was measured by the following procedures: first, low‐density, and very low‐density lipoproteins were precipitated with a reagent containing phosphotungstic acid and magnesium chloride. After this, the precipitate was removed by centrifugation and the remaining HDL‐C in the supernatant was determined with the CHOD‐PAP method. The serum levels of triglycerides and HDL‐C on follow‐up were used as continuous variables in the multivariate analyses.

Information on arterial hypertension, diagnosed by a physician, was gathered from a questionnaire completed in the follow‐up examinations. Arterial hypertension on follow‐up was categorized as no versus yes.

**Figure 2:**
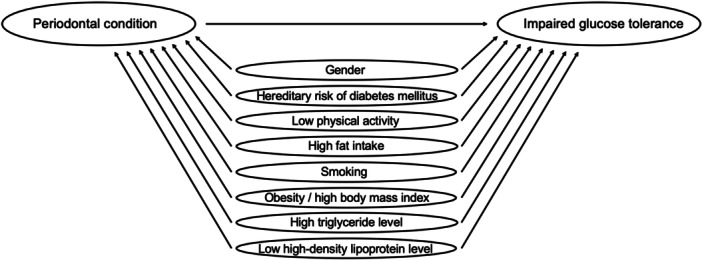
Final model depicting the association between periodontal condition at baseline, covariates, and the presence of impaired glucose tolerance on follow‐up.

The incidence rate ratios and their 95% confidence intervals (CI) were estimated by using the modified Poisson regression with robust error variance (Zou 2004). The selection of the covariates in the multivariate analyses was based on a priori knowledge about the factors involved in glucose metabolism. Missing data in the analyses were managed by creating an additional category “missing data” for those categorical variables with missing observations. Multivariate analyses were tested with and without arterial hypertension as a covariate. It was eventually decided to be left out of the final model (Figure 2) due to a fairly large number of missing observations and to avoid overparameterization, because it had a very minimal effect on the estimates (not more than one‐tenth on few estimates [data not shown]). Multivariate analyses were performed for the total study population and separately among the non‐smoking participants (never smokers and former smokers), and further among the non‐smoking, non‐obese (BMI < 30) participants, as well as among the non‐smoking, non‐obese participants with ≥ 5 missing teeth to minimize bias caused by smoking (Hujoel et al. 2002), excessive body weight, and tooth loss. All statistical analyses were performed using IBM SPSS 23.0.0.0 (SPSS Inc., Chicago, IL, USA) data analysis software.

## 3 Results

Descriptive statistics for the study population according to the categories of periodontal condition at baseline are presented in Table 1, and those according to the presence of IGT on follow‐up are shown in Table 2.

**Table 1:** Basic characteristics of the study population (*n* = 225) according to the categories of periodontal condition in the baseline examinations in 1990–1992.

	Periodontal condition				
	No deepened (≥ 4 mm) periodontal pockets	1–6 sites with deepened periodontal pockets	≥ 7 sites with deepened periodontal pockets	Edentate	Total
Gender (baseline data), *n* (%)					
Males	22 (45)	17 (32)	29 (60)	17 (23)	85 (38)
Females	27 (55)	36 (68)	19 (40)	58 (77)	140 (62)
Level of education (baseline data), *n* (%)					
Basic	34 (70)	39 (73)	33 (69)	61 (81)	167 (74)
Intermediate	5 (10)	6 (11)	8 (17)	2 (3)	21 (9)
High	5 (10)	4 (8)	3 (6)	1 (1)	13 (6)
Missing data	5 (10)	4 (8)	4 (8)	11 (15)	24 (11)
Household income (€/year) (baseline data), *n* (%)					
< 20,400	10 (21)	10 (19)	7 (15)	22 (29)	49 (22)
20,400–30,599	10 (21)	16 (30)	5 (10)	25 (34)	56 (25)
30,600–40,800	10 (21)	13 (25)	16 (33)	10 (13)	49 (22)
> 40,800	11 (22)	7 (13)	14 (29)	5 (7)	37 (16)
Missing data	8 (15)	7 (13)	6 (13)	13 (17)	34 (15)
Mean number of teeth (baseline data), mean (SD)	15 (8.5)	17 (8.1)	19 (7.3)	0	11 (11)
Hereditary risk of diabetes mellitus (follow‐up data), *n* (%)					
Low risk	27 (55)	34 (64)	30 (62)	46 (61)	137 (61)
Increased risk	22 (45)	19 (36)	18 (38)	29 (39)	88 (39)
Physical activity (baseline data), *n* (%)					
High physical activity	39 (80)	42 (79)	30 (63)	60 (80)	171 (76)
Low physical activity	10 (20)	9 (17)	17 (35)	14 (19)	50 (22)
Missing data	0 (0)	2 (4)	1 (2)	1 (1)	4 (2)
Dietary habits (baseline data), *n* (%)					
Healthy diet	32 (65)	33 (62)	24 (50)	37 (49)	126 (56)
Moderately healthy diet	7 (14)	12 (23)	13 (27)	22 (29)	54 (24)
Unhealthy diet	6 (12)	6 (11)	9 (19)	12 (16)	33 (15)
Missing data	4 (8)	2 (4)	2 (4)	4 (5)	12 (5)
Smoking status (follow‐up data), *n* (%)					
Never smoker	24 (49)	31 (58)	12 (25)	28 (37)	95 (42)
Former smoker	22 (45)	20 (38)	29 (60)	38 (51)	109 (49)
Current smoker	3 (6)	2 (4)	7 (15)	9 (12)	21 (9)
Body mass index (BMI) (follow‐up data), *n* (%)					
< 25.0	17 (35)	15 (28)	4 (8)	18 (24)	54 (24)
25.0–29.9	24 (49)	31 (59)	33 (69)	29 (39)	117 (52)
≥ 30.0	8 (16)	7 (13)	11 (23)	28 (37)	54 (24)
Absolute change in BMI during the follow‐up period, *n* (%)					
BMI change negative	20 (41)	7 (13)	11 (23)	13 (17)	51 (23)
0 ≤ BMI change < 2	13 (27)	27 (51)	15 (31)	28 (38)	83 (37)
2 ≤ BMI change < 4	10 (20)	13 (25)	13 (27)	19 (25)	55 (24)
BMI change ≥ 4	6 (12)	6 (11)	9 (19)	15 (20)	36 (16)
BMI (follow‐up data), mean (SD)	26.6 (3.9)	26.4 (3.6)	28.0 (3.2)	29.0 (5.2)	27.6 (4.3)
Serum level of triglycerides (follow‐up data), mean (SD)	1.1 (0.3)	1.3 (0.7)	1.1 (0.4)	1.3 (0.7)	1.2 (0.6)
Serum level of high‐density lipoprotein cholesterol (follow‐up data), mean (SD)	1.7 (0.5)	1.9 (0.5)	1.6 (0.4)	1.7 (0.5)	1.7 (0.5)
Arterial hypertension (follow‐up data), *n* (%)					
No	19 (39)	26 (49)	22 (46)	30 (40)	97 (43)
Yes	19 (39)	19 (36)	22 (46)	31 (41)	91 (41)
Missing data	11 (22)	8 (15)	4 (8)	14 (19)	37 (16)

Abbreviation: SD, standard deviation.

**Table 2:** Basic characteristics of the study population according to the presence of impaired glucose tolerance (IGT) in the follow‐up examinations in 2007–2008.

	IGT^a^		
	Yes	No	Total
Gender (baseline data), *n* (%)			
Males	24 (46)	61 (35)	85 (38)
Females	28 (54)	112 (65)	140 (62)
Level of education (baseline data), *n* (%)			
Basic	41 (78)	126 (73)	167 (74)
Intermediate	4 (8)	17 (9)	21 (9)
High	3 (6)	10 (6)	13 (6)
Missing data	4 (8)	20 (12)	24 (11)
Household income (€/year) (baseline data), *n* (%)			
< 20,400	10 (19)	39 (23)	49 (22)
20,400–30,599	15 (29)	41 (24)	56 (25)
30,600–40,800	14 (27)	35 (20)	49 (22)
> 40,800	9 (17)	28 (16)	37 (16)
Missing data	4 (8)	30 (17)	34 (15)
Mean number of teeth (baseline data), mean (SD)	12 (12)	11 (10)	11 (11)
Periodontal condition (baseline data), *n* (%)			
No deepened (≥ 4 mm) periodontal pockets	6 (12)	43 (25)	49 (22)
1–6 sites with deepened periodontal pockets	13 (25)	40 (23)	53 (24)
≥ 7 sites with deepened periodontal pockets	14 (27)	34 (20)	48 (21)
Edentate	19 (36)	56 (32)	75 (33)
Hereditary risk of diabetes mellitus (follow‐up data), *n* (%)			
Low risk	33 (64)	104 (60)	137 (61)
Increased risk	19 (36)	69 (40)	88 (39)
Physical activity (baseline data), *n* (%)			
High physical activity	36 (69)	135 (78)	171 (76)
Low physical activity	14 (27)	36 (21)	50 (22)
Missing data	2 (4)	2 (1)	4 (2)
Dietary habits (baseline data), *n* (%)			
Healthy diet	23 (44)	103 (59)	126 (56)
Moderately healthy diet	14 (27)	40 (23)	54 (24)
Unhealthy diet	11 (21)	22 (13)	33 (15)
Missing data	4 (8)	8 (5)	12 (5)
Smoking status (follow‐up data), *n* (%)			
Never smoker	11 (21)	84 (48)	95 (42)
Former smoker	37 (71)	72 (42)	109 (49)
Current smoker	4 (8)	17 (10)	21 (9)
Body mass index (BMI) (follow‐up data), *n* (%)			
< 25.0	9 (17)	45 (26)	54 (24)
25.0–29.9	26 (50)	91 (53)	117 (52)
≥ 30.0	17 (33)	37 (21)	54 (24)
Absolute change in BMI during the follow‐up period, *n* (%)			
BMI change negative	8 (15)	43 (25)	51 (23)
0 ≤ BMI change < 2	17 (33)	66 (38)	83 (37)
2 ≤ BMI change < 4	10 (19)	45 (26)	55 (24)
BMI change ≥ 4	17 (33)	19 (11)	36 (16)
BMI (follow‐up data), mean (SD)	29.0 (5.5)	27.2 (3.8)	27.6 (4.3)
Serum level of triglycerides (follow‐up data), mean (SD)	1.4 (0.8)	1.1 (0.5)	1.2 (0.6)
Serum level of high‐density lipoprotein cholesterol (follow‐up data), mean (SD)	1.7 (0.5)	1.8 (0.5)	1.7 (0.5)
Arterial hypertension (follow‐up data), *n* (%)			
No	17 (33)	80 (46)	97 (43)
Yes	27 (52)	64 (37)	91 (41)
Missing data	8 (15)	29 (17)	37 (16)

Abbreviation: SD, standard deviation.

^a^ Post‐load blood glucose value > 7.8 mmol/L after a standard 2‐h oral glucose tolerance test.

The results of the unadjusted analyses of the association of periodontal condition at baseline with the presence of IGT on follow‐up are presented in Table 3, and the results of the multivariate analyses are shown in Table 4. In the multivariate analyses, the participants with deepened (≥ 4 mm) periodontal pockets were found to have a higher likelihood of developing IGT than the periodontally healthy (no deepened periodontal pockets), dentate participants. The risk estimates for the edentate participants for developing IGT were quite close to those for the participants with 1–6 sites with deepened periodontal pockets and weaker than for the participants with ≥ 7 sites with deepened periodontal pockets. The adjusted incidence rate ratios with 95% CI for the participants with 1–6 sites with deepened periodontal pockets, the participants with ≥ 7 sites with deepened periodontal pockets, and the edentate participants (periodontally healthy dentate participants as the reference category) were 1.5 (95% CI, 0.6–4.0), 1.8 (95% CI, 0.7–4.4), and 1.6 (95% CI, 0.6–4.0), respectively. A similar association, albeit somewhat stronger, was found in non‐smokers and among the non‐smoking, non‐obese participants. The observed association was further strengthened when the latter population—that is, the non‐smoking, non‐obese participants—was limited to those with ≥ 5 missing teeth (Table 4).

**Table 3:** Association of the presence of deepened (≥ 4 mm) periodontal pockets and edentulousness at baseline with the presence of impaired glucose tolerance (IGT)^a^ on follow‐up in 2007–2008; unadjusted incidence rate ratios (IRR) with 95% confidence intervals (95% CI).

	IGT			
	Total (Effective *n* = 225)	Non‐smokers^b^ (Effective *n* = 204)	Non‐smokers, non‐obese^c^ (Effective *n* = 156)	Non‐smokers, non‐obese with ≥ 5 missing teeth^d^ (Effective *n* = 149)
Periodontal condition	IRR (95% CI)	IRR (95% CI)	IRR (95% CI)	IRR (95% CI)
No deepened pockets	1.0	1.0	1.0	1.0
1–6 sites with deepened pockets	2.0 (0.8–4.9)	2.2 (0.8–5.7)	2.3 (0.8–6.7)	2.7 (0.8–9.1)
≥ 7 sites with deepened pockets	2.4 (1.0–5.7)	2.9 (1.1–7.5)	2.1 (0.7–6.7)	2.4 (0.7–8.8)
Edentate	2.1 (0.9–4.8)	2.5 (1.0–6.3)	2.0 (0.7–6.1)	2.5 (0.7–8.5)

^a^ Post‐load blood glucose value > 7.8 mmol/L after a standard 2‐h oral glucose tolerance test.

^b^ Current smokers were excluded.

^c^ Current smokers and participants with BMI ≥ 30 were excluded.

^d^ Current smokers and participants with BMI ≥ 30 and < 5 missing teeth were excluded.

**Table 4:** Association of the presence of deepened (≥ 4 mm) periodontal pockets and edentulousness at baseline with the presence of impaired glucose tolerance (IGT)^a^ on follow‐up in 2007–2008; adjusted incidence rate ratios (IRR) with 95% confidence intervals (95% CI).

	IGT			
	Total^b^ (Effective *n* = 225)	Non‐smokers^c^ (Effective *n* = 204)	Non‐smokers, non‐obese^d^ (Effective *n* = 156)	Non‐smokers, non‐obese with ≥ 5 missing teeth^e^ (Effective *n* = 149)
Periodontal condition	IRR (95% CI)	IRR (95% CI)	IRR (95% CI)	IRR (95% CI)
No deepened pockets	1.0	1.0	1.0	1.0
1–6 sites with deepened pockets	1.5 (0.6–4.0)	1.6 (0.6–4.4)	1.7 (0.6–5.3)	2.1 (0.6–7.4)
≥ 7 sites with deepened pockets	1.8 (0.7–4.4)	2.3 (0.9–6.2)	2.2 (0.7–6.7)	2.2 (0.6–8.3)
Edentate	1.6 (0.6–4.0)	1.8 (0.6–4.9)	1.9 (0.6–5.9)	2.4 (0.6–8.7)

^a^ Post‐load blood glucose value > 7.8 mmol/L after a standard 2‐h oral glucose tolerance test.

^b^ Adjusted for gender, level of education, yearly household income, the hereditary risk of diabetes mellitus, physical activity at baseline, dietary habits at baseline, smoking status on follow‐up, BMI on follow‐up, absolute change in BMI during the follow‐up period (continuous variable), and the serum levels of triglycerides and high‐density lipoprotein cholesterol on follow‐up (continuous variables).

^c^ Adjusted for gender, level of education, yearly household income, the hereditary risk of diabetes mellitus, physical activity at baseline, dietary habits at baseline, BMI on follow‐up, absolute change in BMI during the follow‐up period (continuous variable), and the serum levels of triglycerides and high‐density lipoprotein cholesterol on follow‐up (continuous variables). Current smokers were excluded.

^d^ Adjusted for gender, level of education, yearly household income, the hereditary risk of diabetes mellitus, physical activity at baseline, dietary habits at baseline, BMI on follow‐up, absolute change in BMI during the follow‐up period (continuous variable), and the serum levels of triglycerides and high‐density lipoprotein cholesterol on follow‐up (continuous variables). Current smokers and participants with BMI ≥ 30 were excluded.

^e^ Adjusted for gender, level of education, yearly household income, the hereditary risk of diabetes mellitus, physical activity at baseline, dietary habits at baseline, BMI on follow‐up, absolute change in BMI during the follow‐up period (continuous variable), and the serum levels of triglycerides and high‐density lipoprotein cholesterol on follow‐up (continuous variables). Current smokers and participants with BMI ≥ 30 and < 5 missing teeth were excluded.

## 4 Discussion

According to the findings of this cohort study, poor periodontal condition and edentulousness at baseline were associated with the development of IGT over a follow‐up period of approximately 15 years. Furthermore, the association was stronger when smokers and obese participants were excluded.

An obvious strength of this study is the longitudinal study design, which makes it possible to assess the temporal sequence between periodontal condition and IGT. Another strength of this study is that the participants of the cohort were born in the same year, which reduced the cohort effect and the confounding effect regarding age. The long follow‐up period is a strength of this study, but it also has some disadvantages. The obvious strength is that the long follow‐up period is optimal when studying two chronic conditions with a slow onset and progression, such as periodontal disease and IGT. However, a potential risk in such a long follow‐up period is the loss to follow‐up, like in this study. A relevant and justified question is whether this non‐participation leads to attrition bias, which is under‐ or overestimation of the strength of the observed association. Nevertheless, based on the assumption that a relatively large proportion of the dropouts in this cohort were due to age‐related general morbidity and mortality, possible bias can be expected to lead toward underestimation. Another methodological issue regarding the long follow‐up period in this study is that the periodontal measurement was carried out at one time‐ point only: baseline. Changes in the periodontal status of the participants—especially due to possible treatment of active periodontal disease or the lack of regular maintenance therapy of a treated disease—during the follow‐up period could not be assessed comprehensively. The periodontal condition of a subpopulation of the present study was assessed approximately 5 years after the periodontal examinations at baseline. According to the results of Ylöstalo et al. (2010), the periodontal condition of these participants remained on average the same between the baseline and the follow‐up measurements; the correlation was 0.77 both in periodontal pockets with a depth of 4–5 mm and in periodontal pockets 6 mm deep or deeper. However, it can be fairly assumed, that, in addition to improving the periodontal condition, possible periodontal treatment could have also improved the glycemic control of these participants (Simpson et al. 2022), thus leading to the weakening of the observed association between periodontal condition and IGT in this study. In contrast, the progression of the disease due to the lack of treatment or a proper maintenance therapy could have had the opposite effect.

A common concern in non‐experimental studies on diseases with a complex social and biological background is the control of confounding and especially the possibility of residual confounding related to variables difficult to control for. Despite the thorough control of confounding through multivariate models in this study, it is possible that some residual confounding existed due to unidentified and thus uncontrolled factors, or poor operationalization of the known confounding factors. The latter could relate, for instance, to the incomplete measurement of the amount (i.e., pack‐years) and quality of smoking exposure, nutritional factors, or body composition. To overcome residual confounding related to smoking, body weight, and tooth loss, additional analyses were performed among the non‐smoking and the non‐smoking, non‐obese participants, as well as among those non‐smoking, non‐obese participants with ≥ 5 missing teeth.

The explanatory variable is based on only one parameter of periodontal condition—the number of sites with deepened periodontal pockets—rather than clinical attachment loss, alveolar bone loss, bleeding on probing, or the microbial composition of the periodontal pockets, which must also be considered as a limitation.

Additionally, the variability between the diagnostic criteria of IGT and the reproducibility of the status categorized as IGT needs to be taken into consideration, because day‐to‐day variability in blood glucose levels may cause bias in the assessment of IGT, possibly leading to under‐ or overestimation of the incidence of the condition (Hostalek 2019). Finally, the participants in this study were middle‐aged Caucasians, and whether the findings of this study are also generalizable to other ethnic or age groups is unknown.

The findings of this study are in line with earlier studies focusing on the association of deepened periodontal pockets with IGT (Arora et al. 2014; Saito et al. 2004). Arora et al. (2014) reported that clinical measures of periodontitis, namely periodontal pocket depth and attachment loss, were positively associated with IGT in a study population of 1,165 diabetes‐free adults aged 30–80 years. Moreover, Saito et al. (2004). reported that the severity of periodontal condition, measured by pocket depth, was associated with the prevalence of IGT in a study population of 961 Japanese adults aged 40–79 years. In another Japanese study, poor periodontal condition measured by mean alveolar bone loss was reported to be associated with IGT in a study population of 193 men aged 50–55 years (Saito et al. 2005). Somewhat different results were reported by Marugame et al. (2003) and Kowall et al. (2015) According to Marugame et al. (2003) alveolar bone loss was associated with type 2 diabetes mellitus (T2DM), but not with IGT, in a study population of 664 Japanese men aged 46–57 years. Kowall et al. (2015), on the other hand, reported that among 3,086 German adults aged 20–82 years, only a minuscule association was found for mean periodontal pocket depth and mean clinical attachment loss with prediabetes (IFG and IGT). The differences between the findings of these latter studies and the present study may be due to differences in the study design and populations, as well as the definitions and measurements of the outcome and exposure variables and the data collection methods.

In comparison to the findings of a recent study (Myllymäki et al. 2018) on the association of periodontal condition with the development of T2DM based on the same age cohort—although on different subpopulation (only participants with normal glucose metabolism at baseline included in the present study)—it was observed that the association of poor periodontal condition and edentulousness with developing IGT followed an exposure–response pattern almost similarly as the association of poor periodontal condition and edentulousness with developing T2DM. The one exception was that the edentate participants' risk estimates of IGT were slightly higher than the edentate participants' risk estimates of T2DM. The study population of the present study included 11 participants with T2DM on follow‐up (OGTT result ≥ 11.1 mmol/L). As the data were not available on the time of the development of IGT or the transition of IGT to T2DM among these participants, it was reasonable to treat T2DM as a severe form of IGT and include them in the analyses.

An essential question is what explains the association between poor periodontal condition and IGT. One suggested mechanism is bacterial‐induced inflammation and the activation of a pro‐inflammatory cytokine cascade in periodontal tissues. Firm evidence indicates that cytokines originating from the periodontium can enter the systemic circulation (Polak and Shapira 2018), where they play a role in the host immune system by mediating and modulating inflammation (Miranda et al. 2019; Pan, Wang, and Chen 2019). A consequence of low‐grade, chronic systemic inflammation is the underpinning of insulin resistance and pancreatic islet cell apoptosis, leading to insulin deficiency and therefore the progression of glucose intolerance (Fernández‐Real and Pickup 2012). In addition to this mechanistic pathway, free oxygen radicals related to inflammation in periodontal diseases may contribute to systemic inflammation and insulin resistance via oxidative stress (Donath and Shoelson 2011). However, conflicting evidence also exists, and the interindividual variability in both periodontitis and metabolic diseases may be affected by shared genetic and environmental factors (Taylor, Preshaw, and Lalla 2013). A subsequent question is why edentate individuals had an increased likelihood of developing IGT. It can be speculated that this is due to weight gain or an otherwise somewhat unhealthier lifestyle. Moreover, the effect of periodontal disease history and epigenetic alterations on innate immunity cannot be excluded (Cho et al. 2021; Jurdziński, Potempa, and Grabiec 2020).

It has been demonstrated in numerous studies that periodontal treatment has beneficial effects on glucose metabolism among diabetic patients suffering from periodontitis. Although this is based on randomized, controlled clinical trials, and reliable evidence as such, the evidence that periodontitis is causally associated with the development of diabetes is not as certain. Furthermore, the existence of a causal relation in the association of periodontitis with the development of diabetes cannot be confirmed using a randomized study design. Therefore, the assessment of causality between these conditions must rely on the overall judgment of the available scientific evidence.

This study provides further evidence on the possible effects of poor periodontal condition on general health. The findings of this study can be interpreted in a way that individuals with poor periodontal condition or edentate individuals are at increased risk of developing IGT. A previous study based on this cohort demonstrated poor periodontal condition to be associated with the development of diabetes, which lends support to the conception that poor periodontal condition is linked to a prediabetic condition and subsequently diabetes. Periodontitis as a potential risk factor for impaired glucose metabolism and possibly for diabetes development would also mean a potential and scientific basis for the prevention of diabetes and associated conditions.

Keeping in mind the methodological shortcomings and limitations of this study, these cohort data provide additional evidence on the link between periodontal condition and impaired glucose metabolism. Although this is biologically plausible and supported by accumulated evidence, further studies with solid methodology are still required.

## Author Contributions

Ville Myllymäki was involved in research design and research plan in collaboration with co‐authors, conducted the statistical analyses, analyzed the results with co‐authors, wrote the first drafts, and prepared the final and manuscript with the co‐authors. Pekka Ylöstalo and Tuomas Saxlin prepared the research design and research plan in collaboration with the first author, analyzed the results with the first author, edited and revised the manuscript, and prepared the final manuscript with the first author. Anna Liisa Suominen prepared the research design and research plan in collaboration with the first author, analyzed the results with the first author, and prepared the final manuscript with the first author. Matti Knuuttila prepared the final manuscript with the first author. Ulla Rajala designed the study, contributed to data collection, sought approval for the study, prepared the research design and research plan in collaboration with the first author, and prepared the final manuscript with the first author. Sirkka Keinänen‐Kiukaanniemi designed the study, contributed to data collection, sought approval for the study, and prepared the final manuscript with the first author. Sirpa Anttila designed the study, contributed to data collection, sought approval for the study, and prepared the final manuscript with the first author.

## Conflicts of Interest

The authors declare no conflicts of interest.

## Data Availability

The authors have nothing to report.

## References

[cre270023-bib-0001] Allen, E. M. , J. B. Matthews , D. J. O' Halloran , H. R. Griffiths , and I. L. Chapple . 2011. “Oxidative and Inflammatory Status in Type 2 Diabetes Patients With Periodontitis.” Journal of Clinical Periodontology 38, no. 10: 894–901. 10.1111/j.1600-051x.2011.01764.x. 21883360

[cre270023-bib-0002] Andriankaja, O. M. , K. Joshipura , F. Muñoz , B. A. Dye , F. B. Hu , and C. M. Pérez . 2022. “Impact of Impaired Glucose Metabolism on Periodontitis Progression Over Three Years.” Dentistry Journal 10, no. 1: 1–12. 10.3390/dj10010010. PMC8774522 35049608

[cre270023-bib-0003] Arora, N. , P. N. Papapanou , M. Rosenbaum , D. R. Jacobs Jr. , M. Desvarieux , and R. T. Demmer . 2014. “Periodontal Infection, Impaired Fasting Glucose, and Impaired Glucose Tolerance: Results From the Continuous National Health and Nutrition Examination Survey 2009–2010.” Journal of Clinical Periodontology 41, no. 7: 643–652. 10.1111/jcpe.12258. 24708451 PMC4072528

[cre270023-bib-0004] Bullon, P. , J. M. Morillo , M. C. Ramirez‐Tortosa , J. L. Quiles , H. N. Newman , and M. Battino . 2009. “Metabolic Syndrome and Periodontitis: Is Oxidative Stress a Common Link?” Journal of Dental Research 88, no. 6: 503–518. 10.1177/0022034509337479. 19587154

[cre270023-bib-0005] Cederberg, H. , T. Saukkonen , M. Laakso , et al. 2010. “Postchallenge Glucose, A1C, and Fasting Glucose as Predictors of Type 2 Diabetes and Cardiovascular Disease: A 10‐Year Prospective Cohort Study.” Diabetes Care 33, no. 9: 2077–2083. 10.2337/dc10-0262. 20573752 PMC2928368

[cre270023-bib-0006] Chapple, I. L. , and J. B. Matthews . 2007. “The Role of Reactive Oxygen and Antioxidant Species in Periodontal Tissue Destruction.” Periodontology 2000 43, no. 1: 160–232. 10.1111/j.1600-0757.2006.00178.x. 17214840

[cre270023-bib-0007] Cho, Y. D. , W. J. Kim , H. M. Ryoo , et al. 2021. “Current Advances of Epigenetics in Periodontology From ENCODE Project: A Review and Future Perspectives.” Clinical Epigenetics 13, no. 1: 92. 10.1186/s13148-021-01074-w. 33902683 PMC8077755

[cre270023-bib-0008] Choi, Y. H. , R. E. McKeown , E. J. Mayer‐Davis , A. D. Liese , K. B. Song , and A. T. Merchant . 2011. “Association Between Periodontitis and Impaired Fasting Glucose and Diabetes.” Diabetes Care 34, no. 2: 381–386. 10.2337/dc10-1354. 21216848 PMC3024353

[cre270023-bib-0009] Declaration of Helsinki . 1976. “Recommendations Guiding Medical Doctors in Biomedical Research Involving Human Subjects.” Ugeskr Laeger 138, no. 7: 399–400. https://www.wma.net/wp-content/uploads/2016/11/DoH-Oct1975.pdf. 1251519

[cre270023-bib-0010] Díaz‐Redondo, A. , C. Giráldez‐García , L. Carrillo , et al. 2015. “Modifiable Risk Factors Associated With Prediabetes in Men and Women: A Cross‐Sectional Analysis of the Cohort Study in Primary Health Care on the Evolution of Patients With Prediabetes (PREDAPS‐Study).” BMC Family Practice 16, no. 5: 5. 2015. 10.1186/s12875-014-0216-3. 25609029 PMC4316391

[cre270023-bib-0011] Donath, M. Y. , and S. E. Shoelson . 2011. “Type 2 Diabetes as an Inflammatory Disease.” Nature Reviews. Immunology 11, no. 2: 98–107. 10.1038/nri2925. 21233852

[cre270023-bib-0012] Fernández‐Real, J. M. , and J. C. Pickup . 2012. “Innate Immunity, Insulin Resistance and Type 2 Diabetes.” Diabetologia 55, no. 2: 273–278. 10.1007/s00125-011-2387-y. 22124608

[cre270023-bib-0013] Greiner, G. G. , K. M. F. Emmert‐Fees , J. Becker , et al. 2020. “Toward Targeted Prevention: Risk Factors for Prediabetes Defined By Impaired Fasting Glucose, Impaired Glucose Tolerance and Increased HbA1c in the Population‐Based KORA Study From Germany.” Acta Diabetologica 57, no. 12: 1481–1491. 10.1007/s00592-020-01573-x. 32748175 PMC7591423

[cre270023-bib-0014] Hostalek, U. 2019. “Global Epidemiology of Prediabetes— Present and Future Perspectives.” Clinical Diabetes and Endocrinology 5, no. 5: 5. 2019. 10.1186/s40842-019-0080-0. 31086677 PMC6507173

[cre270023-bib-0015] Hujoel, P. P. , M. Drangsholt , C. Spiekerman , and T. A. DeRouen . 2002. “Periodontitis‐Systemic Disease Associations in the Presence of SmokingCausal or Coincidental?” Periodontology 2000 30, no. 1: 51–60. 10.1034/j.1600-0757.2002.03005.x. 12236895

[cre270023-bib-0016] Jurdziński, K. T. , J. Potempa , and A. M. Grabiec . 2020. “Epigenetic Regulation of Inflammation in Periodontitis: Cellular Mechanisms and Therapeutic Potential.” Clinical Epigenetics 12, no. 1: 186. 10.1186/s13148-020-00982-7. 33256844 PMC7706209

[cre270023-bib-0017] Kowall, B. , B. Holtfreter , H. Völzke , et al. 2015. “Pre‐Diabetes and Well‐Controlled Diabetes Are Not Associated With Periodontal Disease: The SHIP Trend Study.” Journal of Clinical Periodontology 42, no. 5: 422–430. 10.1111/jcpe.12391. 25808753

[cre270023-bib-0018] Mansourian, M. , A. Yazdani , E. Faghihimani , A. Aminorraya , M. Amini , and T. Jafari‐Koshki . 2020. “Factors Associated With Progression to Pre‐Diabetes: A Recurrent Events Analysis.” Eating and Weight Disorders: EWD 25, no. 1: 135–141. 10.1007/s40519-018-0529-7. 29931448

[cre270023-bib-0019] Marugame, T. , H. Hayasaki , K. Lee , H. Eguchi , and S. Matsumoto . 2003. “Alveolar Bone Loss Associated With Glucose Tolerance in Japanese Men.” Diabetic Medicine 20, no. 9: 746–751. 10.1046/j.1464-5491.2003.00989.x. 12925056

[cre270023-bib-0020] Meyle, J. , and I. Chapple . 2015. “Molecular Aspects of the Pathogenesis of Periodontitis.” Periodontology 2000 69, no. 1: 7–17. 10.1111/prd.12104. 26252398

[cre270023-bib-0021] Miranda, T. S. , S. L. Heluy , D. F. Cruz , et al. 2019. “The Ratios of Pro‐Inflammatory to Anti‐Inflammatory Cytokines in the Serum of Chronic Periodontitis Patients With and Without Type 2 Diabetes And/Or Smoking Habit.” Clinical Oral Investigations 23, no. 2: 641–650. 10.1007/s00784-018-2471-5. 29737428

[cre270023-bib-0022] Myllymäki, V. , T. Saxlin , M. Knuuttila , et al. 2018. “Association Between Periodontal Condition and the Development of Type 2 Diabetes Mellitus—Results From a 15‐year Follow‐Up Study.” Journal of Clinical Periodontology 45, no. 11: 1276–1286. 10.1111/jcpe.13005. 30133880

[cre270023-bib-0023] Nesse, W. , F. Abbas , I. van der Ploeg , F. K. Spijkervet , P. U. Dijkstra , and A. Vissink . 2008. “Periodontal Inflamed Surface Area: Quantifying Inflammatory Burden.” Journal of Clinical Periodontology 35, no. 8: 668–673. 10.1111/j.1600-051x.2008.01249.x. 18564145

[cre270023-bib-0024] Nesse, W. , A. Linde , F. Abbas , et al. 2009. “Dose‐Response Relationship Between Periodontal Inflamed Surface Area and HbA1c in Type 2 Diabetics.” Journal of Clinical Periodontology 36, no. 4: 295–300. 10.1111/j.1600-051x.2009.01377.x. 19426175

[cre270023-bib-0025] Pan, W. , Q. Wang , and Q. Chen . 2019. “The Cytokine Network Involved in the Host Immune Response to Periodontitis.” International Journal of Oral Science 11, no. 3: 30. 10.1038/s41368-019-0064-z. 31685798 PMC6828663

[cre270023-bib-0026] Polak, D. , and L. Shapira . 2018. “An Update on the Evidence for Pathogenic Mechanisms That May Link Periodontitis and Diabetes.” Journal of Clinical Periodontology 45, no. 2: 150–166. 10.1111/jcpe.12803. 29280184

[cre270023-bib-0027] Rajala, U. , S. Keinänen‐Kiukaanniemi , A. Uusimäki , K. Reijula , and S. L. Kivelä . 1995. “Prevalence of Diabetes Mellitus and Impaired Glucose Tolerance in a Middle‐Aged Finnish Population.” Scandinavian Journal of Primary Health Care 13, no. 3: 222–228. 10.3109/02813439508996765. 7481176

[cre270023-bib-0028] Saito, T. , Y. Shimazaki , Y. Kiyohara , et al. 2004. “The Severity of Periodontal Disease Is Associated With the Development of Glucose Intolerance in Non‐Diabetics: The Hisayama Study.” Journal of Dental Research 83, no. 6: 485–490. 10.1177/154405910408300610. 15153457

[cre270023-bib-0029] Saito, T. , Y. Shimazaki , Y. Kiyohara , et al. 2005. “Relationship Between Obesity, Glucose Tolerance, and Periodontal Disease in Japanese Women: The Hisayama Study.” Journal of Periodontal Research 40, no. 4: 346–353. 10.1111/j.1600-0765.2005.00813.x. 15966913

[cre270023-bib-0030] Sakki, T. K. , M. L. Knuuttila , S. S. Vimpari , and M. S. Hartikainen . 1995. “Association of Lifestyle With Periodontal Health.” Community Dentistry and Oral Epidemiology 23, no. 3: 155–158. 10.1111/j.1600-0528.1995.tb00220.x. 7634770

[cre270023-bib-0031] Saleem, S. M. , S. S. Jan , I. Haq , and S. M. S. Khan . 2019. “Identification of Risk Factors Affecting Impaired Glucose Metabolism Among the Adult Population of District Srinagar.” Diabetes & Metabolic Syndrome 13, no. 2: 1047–1051. 10.1016/j.dsx.2019.01.023. 31336442

[cre270023-bib-0032] Simpson, T. C. , J. E. Clarkson , H. V. Worthington , et al. 2022. “Treatment of Periodontitis for Glycaemic Control in People With Diabetes Mellitus.” Cochrane Database of Systematic Reviews 2022: CD004714. 10.1002/14651858.cd004714.pub4. PMC9009294 35420698

[cre270023-bib-0033] Song, T. J. , Y. Chang , J. Jeon , and J. Kim . 2021. “Oral Health and Longitudinal Changes in Fasting Glucose Levels: A Nationwide Cohort Study.” PLoS One 16, no. 6: e0253769. 2021. 10.1371/journal.pone.0253769. 34185817 PMC8241120

[cre270023-bib-0034] Taylor, J. J. , P. M. Preshaw , and E. Lalla . 2013. “A Review of the Evidence for Pathogenic Mechanisms That May Link Periodontitis and Diabetes.” Journal of Periodontology 84, no. 4S: 113–134. 10.1902/jop.2013.134005. 23631573

[cre270023-bib-0035] UNESCO . 1997. International Standard Classification of Education: ISCED. https://unstats.un.org/unsd/classifications/family/detail/223.

[cre270023-bib-0036] WHO & IDF . 2006. “Definition and Diagnosis of Diabetes Mellitus and Intermediate Hyperglycaemia.” https://www.who.int/publications/i/item/definition-and-diagnosis-of-diabetes-mellitus-and-intermediate-hyperglycaemia.

[cre270023-bib-0037] World Health Organization . 1985. "Diabetes Mellitus: Report of a WHO Study Group.” World Health Organ Tech Rep Ser. no. 727. 1–113. https://iris.who.int/handle/10665/39592. 3934850

[cre270023-bib-0038] Ylöstalo, P. , S. Anttila , U. Rajala , et al. 2010. “Periodontal Infection and Subclinical Atherosclerosis: The Role of High‐Density Lipoprotein as a Modifying Factor.” Journal of Clinical Periodontology 37, no. 7: 617–624. 10.1111/j.1600-051X.2010.01572.x. 20528962

[cre270023-bib-0039] Zou, G. 2004. “A Modified Poisson Regression Approach to Prospective Studies With Binary Data.” American Journal of Epidemiology 159, no. 7: 702–706. 10.1093/aje/kwh090. 15033648

